# Generalization of finetuned transformer language models to new clinical contexts

**DOI:** 10.1093/jamiaopen/ooad070

**Published:** 2023-08-16

**Authors:** Kevin Xie, Samuel W Terman, Ryan S Gallagher, Chloe E Hill, Kathryn A Davis, Brian Litt, Dan Roth, Colin A Ellis

**Affiliations:** Department of Bioengineering, University of Pennsylvania, Philadelphia, Pennsylvania 19104, USA; Center for Neuroengineering and Therapeutics, University of Pennsylvania, Philadelphia, Pennsylvania 19104, USA; Department of Neurology, University of Michigan, Ann Arbor, Michigan 48109, USA; Center for Neuroengineering and Therapeutics, University of Pennsylvania, Philadelphia, Pennsylvania 19104, USA; Perelman School of Medicine, University of Pennsylvania, Philadelphia, Pennsylvania 19104, USA; Department of Neurology, University of Michigan, Ann Arbor, Michigan 48109, USA; Center for Neuroengineering and Therapeutics, University of Pennsylvania, Philadelphia, Pennsylvania 19104, USA; Perelman School of Medicine, University of Pennsylvania, Philadelphia, Pennsylvania 19104, USA; Department of Neurology, University of Pennsylvania, Philadelphia, Pennsylvania 19104, USA; Department of Bioengineering, University of Pennsylvania, Philadelphia, Pennsylvania 19104, USA; Center for Neuroengineering and Therapeutics, University of Pennsylvania, Philadelphia, Pennsylvania 19104, USA; Perelman School of Medicine, University of Pennsylvania, Philadelphia, Pennsylvania 19104, USA; Department of Neurology, University of Pennsylvania, Philadelphia, Pennsylvania 19104, USA; Department of Computer and Information Science, University of Pennsylvania, Philadelphia, Pennsylvania 19104, USA; Center for Neuroengineering and Therapeutics, University of Pennsylvania, Philadelphia, Pennsylvania 19104, USA; Perelman School of Medicine, University of Pennsylvania, Philadelphia, Pennsylvania 19104, USA; Department of Neurology, University of Pennsylvania, Philadelphia, Pennsylvania 19104, USA

**Keywords:** electronic health records, natural language processing, clinical informatics, epilepsy

## Abstract

**Objective:**

We have previously developed a natural language processing pipeline using clinical notes written by epilepsy specialists to extract seizure freedom, seizure frequency text, and date of last seizure text for patients with epilepsy. It is important to understand how our methods generalize to new care contexts.

**Materials and methods:**

We evaluated our pipeline on unseen notes from nonepilepsy-specialist neurologists and non-neurologists without any additional algorithm training. We tested the pipeline out-of-institution using epilepsy specialist notes from an outside medical center with only minor preprocessing adaptations. We examined reasons for discrepancies in performance in new contexts by measuring physical and semantic similarities between documents.

**Results:**

Our ability to classify patient seizure freedom decreased by at least 0.12 agreement when moving from epilepsy specialists to nonspecialists or other institutions. On notes from our institution, textual overlap between the extracted outcomes and the gold standard annotations attained from manual chart review decreased by at least 0.11 F_1_ when an answer existed but did not change when no answer existed; here our models generalized on notes from the outside institution, losing at most 0.02 agreement. We analyzed textual differences and found that syntactic and semantic differences in both clinically relevant sentences and surrounding contexts significantly influenced model performance.

**Discussion and conclusion:**

Model generalization performance decreased on notes from nonspecialists; out-of-institution generalization on epilepsy specialist notes required small changes to preprocessing but was especially good for seizure frequency text and date of last seizure text, opening opportunities for multicenter collaborations using these outcomes.

## INTRODUCTION

The electronic health record (EHR) holds a wealth of clinical information with both breadth, covering the potentially hundreds of thousands of patients that visit a health system, and depth, tracking individual patients over their time within the system. Thus, the EHR is especially well suited for retrospective clinical studies: Clinicians can use the EHR to follow patient cohorts backwards through time, often at less cost and time than prospective clinical studies.^1–4^ Furthermore, EHRs from multiple institutions can be pooled together to drive large-scale multicenter research.^5^

Much of the information in the EHR is stored in the raw text of clinic notes written by healthcare providers during and following an office visit or procedure. Natural language processing (NLP), a field of machine learning dedicated to training machines to understand human language, can be used to mine information from these clinic notes. However, text is inherently heterogenous—each provider has their own writing style, each discipline their own vocabulary and knowledge, and each institution their own format and biases.^6^^,^^7^ These differences necessitate methods capable of adapting to previously unseen care settings with minimal loss of accuracy and preclude rules-based and traditional machine learning-based NLP approaches.^8^ In contrast, Transformer language models, including *OpenAI’s* ChatGPT, have shown remarkable generalizability across multiple tasks and domains, often with minimal manual intervention.^9–12^ We have previously developed and validated an NLP pipeline that finetuned and applied BERT-like transformer models to extract 3 critical outcome measures—seizure freedom, seizure frequency text, and time since last seizure text—from clinic notes describing patients with epilepsy,^13^^,^^14^ and have used it to better characterize long-term epilepsy outcome dynamics.^15^ Although our methods have achieved up to human-like performance within internal test data, we had yet to evaluate our methods’ ability to generalize to new contexts.

Here, we expand upon our previous study and investigate how well our pipeline extracts key epilepsy outcome measures from unseen data. We first tested within-institution generalizability by evaluating performance on clinic notes from nonepilepsy departments without any additional algorithm finetuning or modification; we expected these new notes to have similar formatting as the previously studied epilepsy notes, but potentially new styles, vocabulary, and context. We then tested out-of-institution generalizability by evaluating performance on epileptologist notes from the University of Michigan, again without any additional model finetuning, but with modifications to preprocessing. We expected these out-of-institution notes to have similar vocabulary and context as our epilepsy notes, but potentially different styles and formats. We discuss potential future improvements and possibilities for use in multicenter research.

## METHODS

### Penn document curation and annotation

Our source dataset included 78 844 progress notes for patients with epilepsy who visited the University of Pennsylvania Health System between January 1, 2015, and December 31, 2018. In this manuscript, we refer to neurologists who were specialized in the management of epilepsy (epilepsy specialists) as “Epileptologists,” neurologists who were not epilepsy specialists as “Neurologists,” and all other clinical providers as “Non-neurologists.” As previously described,^13^ we had annotated 1000 notes written by epileptologists for 3 epilepsy outcome measures: seizure freedom, seizure frequency, and date of last seizure. To summarize the annotation protocol, annotators marked the first span of text that suggested a patient was seizure free or having recent seizures, and all spans of text that described the patient’s seizure frequency or date of last seizure. Documents were annotated in triplicate, and merged through majority voting with manual adjudication of disagreements. We used 700 annotated notes as training data and 300 notes as test data. Here, we created 2 new datasets to evaluate generalization performance within our institution. We identified 100 notes written by neurologists, and 100 notes written by non-neurologists, that contained the word “seizure” within them. Two authors, KX and CAE, each manually annotated these 200 notes for the same 3 epilepsy outcome measures, using the same annotation protocol as in the previous study. As before, we merged annotations and resolved disagreements through manual adjudication. The number of notes chosen was based upon our prior results indicating that model performance plateaued when trained on 100 sample notes.

### Penn pipeline preprocessing and evaluation

Our NLP pipeline was previously described in detail.^13^^,^^14^ Briefly, we preprocessed the notes by isolating the History of Present Illness and/or Interval History sections from the full note text. We then truncated these sections to 1506 characters to fit within the token limit of our BERT models and formatted them for use with the Huggingface Python library.^16^ We ran these preprocessed paragraphs into our NLP pipeline with 5 random seeds, and extracted 5 sets of seizure freedom, seizure frequency text, and date of last seizure text from each datum. Our pipeline treated seizure freedom as a trinary classification task, where the patient was either seizure free, having seizures, or no classification could be made with the given information. We classified visits as “seizure free” if the patient had not had a seizure in the past year or since their last visit, whichever was more recent. We extracted seizure frequency text and date of last seizure text through a text extraction task, where the models attempted to find the span of text where the outcome measure was described (ie, “patient had 3 seizures in the last 6 months”). We defined a “seizure frequency” as any statement from which one could compute a positive quantity representing the number of seizures per unit time (ie, “3 seizures in the last 6 months”); we defined a “date of last seizure” as any statement from which a date can be assigned to (ie, “last seizure was in 2015”).

### Contributors to generalization performance

We next tried to identify contributors to generalization performance. We hypothesized that changes in performance were due to 2 factors. First, there may be differences between how epileptologists and nonepileptologists present relevant information, specifically in how they write it. For example, an epileptologist may write “patient reports focal impaired awareness seizure last Sunday, no convulsion,” while a non-neurologist may write “patient reports a seizure on Sunday,” thus missing the specific seizure type. In this study, we called the sentences containing such germane information the “Kernel” of the passage; the Kernel is also the target of our text extraction tasks.

Second, there may be differences in how the contextual information surrounding the Kernel is presented. An epileptologist’s note largely contains information related to epilepsy and a patient’s epilepsy history. However, a neurologist note may mention a patient’s epilepsy but may also discuss other neurological disorders; a non-neurologist’s note may briefly mention epilepsy or seizures, before proceeding to other topics, like cardiovascular problems for a cardiologist. We call this surrounding contextual information the “Surrounding Context.”

We quantified these differences through the Cosine Similarity and Levenshtein Similarity metrics.^17^ Cosine similarity, when combined with sentence embeddings, measures how similar in meaning 2 sentences are to each other—a semantic distance that estimates how different in context and meaning 2 sentences are. Sentence embeddings use neural networks to map sentences into high dimensional spaces such that 2 similar concepts are located near each other.^18^ Levenshtein similarity measures how many insertions, deletions, or substitutions are required to change one sentence to another—a “physical” distance that estimates for how differently written 2 sentences are.

Using these metrics, we computed the pairwise similarity of each note in our 3 categories of within-institution test sets (300 epileptologist, 100 neurologist, and 100 non-neurologist notes) to each of the 700 epileptologist notes in the training set. For each pair of notes, we computed 4 separate similarity measures: Levenshtein-kernel, Levenshtein-context, cosine-kernel, and cosine-context. We generated the sentence embeddings for the cosine similarity metric using pretrained sentence-transformers. ^19^ As a reference for comparison, we also computed the similarity of each pair of notes within the training set, using the same methods.

### Michigan document curation, preprocessing, and evaluation

We gathered 100 random outpatient neurology visit notes, written by an epileptologist, of adult patients with epilepsy who visited the University of Michigan medical center between 2015 and 2019.^20^ These notes were formatted differently than Penn notes, including, for example, notes that had no explicit sections for “History of Present Illness” and “Interval History.” We modified our preprocessing steps to search for the following keywords and phrases indicative of the beginning of epilepsy history: “the patient was last seen,” “interval history,” “interval events,” “hpi,” and “history of present illness.” We took 1506 characters, beginning with the sentence that contained the identified keywords or phrases. If no keywords were found, we took the first 1506 characters of the document. We formatted the extracted paragraphs for Huggingface and ran them through our pipeline. All code and analysis that used these notes from the University of Michigan were run from within that institution in a federated manner; we did not transfer data or merge datasets between institutions to protect patient health information. We did not perform textual similarity analysis on these notes for the same reason.

### Statistical analysis

To assess inter-rater reliability of human annotations for the 100 neurologist and 100 non-neurologist notes, we used Cohen’s Kappa^21^ for seizure freedom annotations, and the text overlap F_1_ score for seizure frequency text and date of last seizure text annotations. The Cohen’s Kappa (κ) score measures the agreement between annotators offset by their expected chance agreement; a κ greater than 0.61 indicates “substantial” agreement, and greater than 0.81 indicates “near perfect” agreement.^22^ The F_1_ score measures text overlap, accounting for both the presence and order of characters and/or words, such that a F_1_ of 1.0 indicates identical spans of texts. We calculated F_1_ in 2 ways: A paired F_1_, which shows overlap when 2 annotators agree on some amount of text, and an overall F_1_, which includes all annotated spans of text of an outcome measure in each note, such as when one annotator marked a span of text but the other did not. We compared these results to the same metrics of inter-rater reliability from our annotations of the epileptologists notes, which were previously reported.^13^

Next, we matched the pipeline’s predictions against the gold-standard annotations and used percent agreement to evaluate classification of seizure freedom, and text overlap F_1_ to evaluate text extraction performance. We defined agreement as the number of times the pipeline was able to correctly capture the relevant information in the ground truth, divided by the total number of examples; in classification tasks, agreement is equivalent to accuracy. However, we use agreement even in classification tasks to maintain a single concise metric of model performance across all our evaluations. If multiple correct answers existed in the gold-standard, we took the most-well-matched answer. We then tested for differences between pipeline performances on the original 300 epileptologist validation notes, and the new 100 neurologist, and 100 non-neurologist notes using 2-sided Mann-Whitney *U*-tests with the null hypothesis that the performances on the 3 datasets were identical (came from the same distribution). As a reference, on our epileptologist validation notes, our human annotators previously attained a median classification agreement of 0.92 (range 0.65–0.98), and median text extraction F_1_ of 0.89 for seizure frequency text (range 0.73–0.96) and 0.78 for date of last seizure text (range 0.60–0.92).^13^

To explore how similarity measures influenced model performance, we tested for univariate associations between these measures and classification correctness. For each document in a testing set, we calculated the mean of the pairwise similarity to each of the 700 training notes. We did this separately for each of the 4 similarity measures (Levenshtein-kernel, Levenshtein-context, cosine-kernel, cosine-context), such that each note in the 3 test sets had 4 separate mean similarity scores. We then split these measures by whether the model made correct seizure freedom predictions on the testing documents and used 2-sided Mann-Whitney *U*-tests to compare the similarity to training set in the correct versus incorrect distributions. We performed a similar analysis on the text extraction tasks, comparing the similarity of testing documents to the training set in cases where the F_1_ score was at least 0.50 verses less than 0.50.

Next, we compared the distributions of pairwise similarity to the training set for our 3 test sets (epileptologist, neurologist, non-neurologist) and for each of the 4 similarity metrics. We used Q-Q plots to compare each distribution against the reference distribution of pairwise similarity scores within the training set. As suggested in Lin et al, we forwent statistical tests and *P*-values when comparing these distributions, as *P*-values can become arbitrarily small with large sample sizes (ie, there are 700^2^ pairwise comparisons within the training set alone).^23^

To evaluate pipeline performance on notes from Michigan, we calculated percent agreement scores for all 3 outcome measures. We used agreement instead of the text overlap F_1_ score for evaluating the extraction of seizure frequency and date of last seizure texts because the notes from Michigan lacked annotations. Instead, author SWT manually reviewed the pipeline’s output for agreement with the corresponding relevant information in each note under our definitions of seizure freedom, frequency, and date of last seizure. We used a single seed instead of 5 to minimize the amount of manual review.

We used Python with the Huggingface Transformers, Huggingface Datasets, Sentence-Transformers, Numpy, Pandas, Scipy, Statsmodels, and Levenshtein packages. Our models and code are on the Huggingface Hub and Github at https://huggingface.co/CNT-UPenn, and https://github.com/penn-cnt/generalization_of_nlp_in_clinical_contexts, respectively. We do not share our data to protect patient privacy, as our data are not deidentified.

## RESULTS

### Inter-rater reliability and gold-standard annotations

Inter-rater agreement of 2 human annotators on notes from epileptologists, neurologists, and non-neurologists are shown in Table 1. Overall F_1_ scores were lower than paired F_1_ scores, indicating some missing annotated spans between annotators in the text extraction tasks and emphasizing the importance of our manual annotation adjudication. More critically, there was no major difference in human annotators’ interpretation of these categories of notes. The underlying statistics of the adjudicated annotations were different between the epileptologist, neurologist, and non-neurologist notes (Table 2). Overall, documentation of seizure freedom, frequency, and date of last seizure became sparser as one stepped away from epileptology.

**Table 1.** Annotator agreement

	Epileptologist notes (mean)^a^	Neurologist notes	Non-neurologist notes
Seizure Freedom Classification Agreement (κ)	0.82	0.82	0.74
Overall Extraction Agreement (F_1_)	0.44	0.47	0.42
Paired Extraction Agreement (F_1_)	0.79	0.85	0.93

a From our previous study.^13^

**Table 2.** Statistics of notes from the University of Pennsylvania

Gold standard annotations	Epileptologist (*n* = 1000, 700 training, 300 validation)^a^	Neurologist (*n* = 100)	Non-neurologist (*n* = 100)
Classification			
Seizure-free	30%	33%	30%
Not seizure-free	62%	47%	35%
Unclassified	8%	20%	35%
Note contained seizure frequency text	36%	14%	7%
Note contained date of last seizure text	50%	48%	36%

a From our previous study.^13^

### Pipeline performance across author specialties

**Figure 1.**
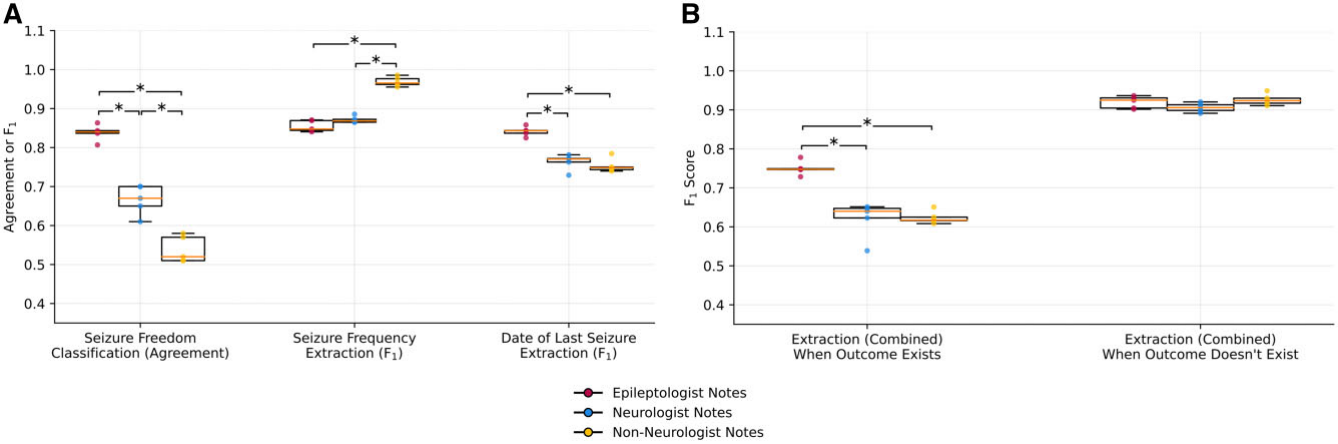
Generalization of the NLP pipeline on classifying patient seizure freedom and extracting seizure frequency text and date of last seizure text. (A) Classification agreement with ground truth annotations and text overlap F_1_ scores on respective outcome measures on epileptologist (red dots), neurologist (blue dots), and non-neurologist (yellow dots) notes using random 5 seeds. Significant differences with *P* < .0167 were found using 2-sided Mann-Whitney *U*-tests and are marked with an asterisk. (B) Pipeline text overlap F_1_ scores, separated by whether an outcome measure was present in the note, combining the seizure frequency and date of last seizure text extraction tasks.

This analysis compared notes from epileptologists, neurologists, and non-neurologists within a single institution. For the classification of seizure freedom, agreement between algorithm predictions and human annotations decreased by 0.17 between epileptologists and neurologists, and decreased further by 0.12 between neurologists and non-neurologists. For extraction of seizure frequency text, model performance (F_1_) increased by 0.12 between epileptologists and non-neurologists, and by 0.09 between neurologists and non-neurologists. For extraction of date of last seizure text, model performance (F_1_) decreased by 0.07 between epileptologists and neurologists, and decreased by 0.09 between epileptologists and non-neurologists (Figure 1A).

Performance of these text extraction tasks varied according to whether an answer was present in the note (Figure 1B). The algorithm detected that no answer was present equally well across specialties. However, when an answer did exist, the pipeline’s ability to match the answer’s text significantly decreased for neurologists and non-neurologists compared to epileptologists. Therefore, the apparent increase in model performance for seizure frequency text was likely driven by the higher frequency of notes with no answer across specialties.

### Model performance and similarity to training set notes

**Figure 2.**
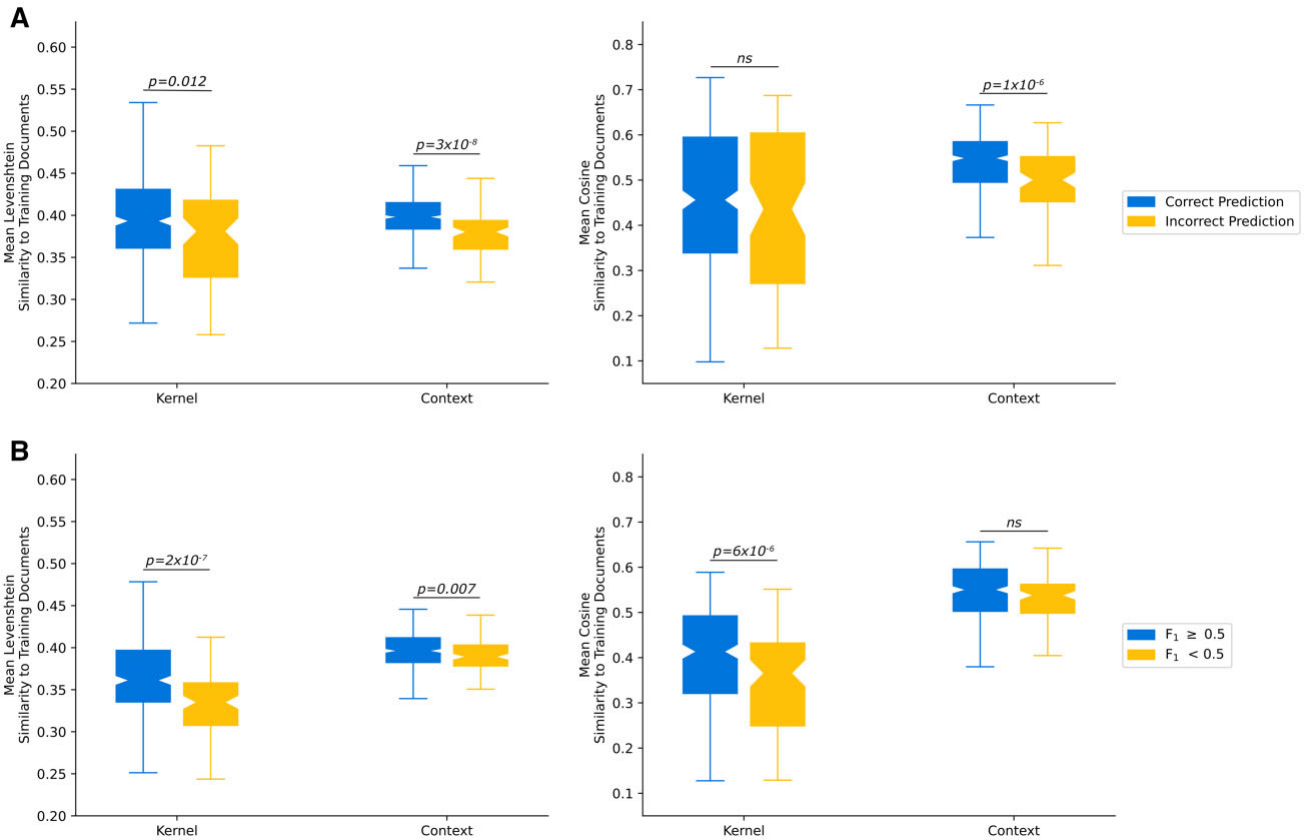
Comparison of text similarity measures. (A) Text similarity of the Kernels and Contexts, stratified by whether the model made a correct seizure freedom classification. (B) Text similarity, stratified by if the model made a prediction with at least 0.5 text overlap F_1_ with the ground truth annotation.

We hypothesized that notes where the pipeline performed better were more similar to the training set than notes classified incorrectly. For the classification of seizure freedom task, this hypothesis was supported for 3 of 4 similarity measures (Figure 2A): Levenshtein similarity of kernels (*P* = .012), Levenshtein similarity of contexts (*P* = 3×10^−8^), and cosine similarity of contexts (*P* = 1×10^−6^). Meanwhile, for our 2 text extraction tasks, this hypothesis was also supported for 3 of 4 similarity measures (Figure 2B): Levenshtein similarity of kernels (*P* = 2×10^−7^), the cosine similarity of the kernels (*P* = 6×10^−6^), and the Levenshtein similarity of the context (*P* = .007). These findings suggest that the pipeline performed better when notes were more similar to the training set. Specifically, for the text classification task, this effect was stronger for the surrounding context than for the kernel (target) text. Meanwhile, in the text extraction task, the opposite can be seen—this effect was stronger for the kernel text than the surrounding context.

**Figure 3.**
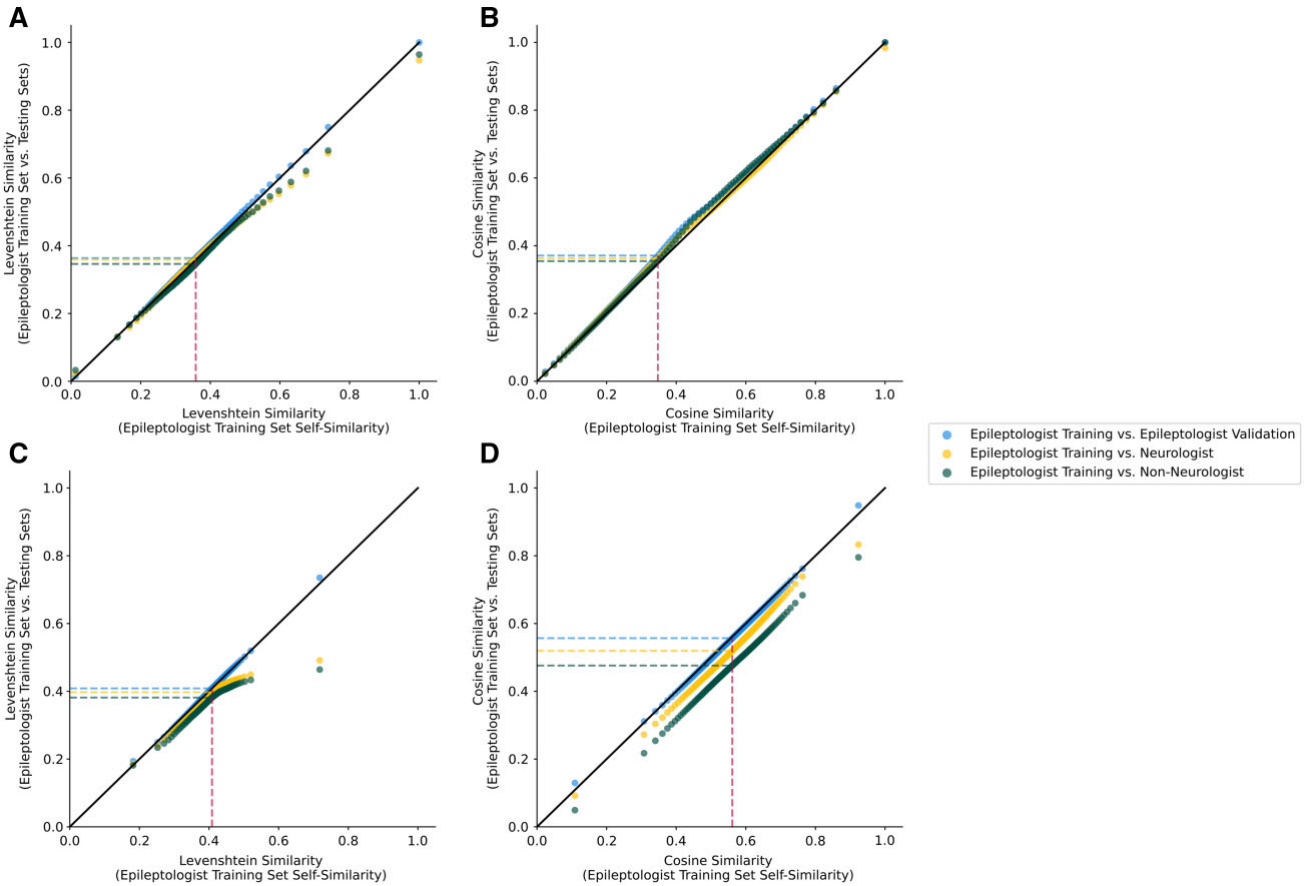
Q-Q plots of the textual similarity in the reference epileptologist training set (*x*-axis) against the epileptologist validation set (blue), neurologist set (yellow), and non-neurologist set (green) on the *y*-axis. The black line indicates the identity line *y* = *x*. The dashed lines indicate the medians of each distribution: similarity within the reference epileptologist training set (red), epileptologist validation set (blue), neurologist set (yellow), and non-neurologist set (green). Panels: (A) Levenshtein similarity of kernels. (B) Cosine similarity of kernels. (C) Levenshtein similarity of surrounding contexts. (D) Cosine similarity of surrounding contexts.

Next, we examined the distributions of similarity to the training set for our 3 test sets: epileptologists, neurologists, and non-neurologists. We used Q-Q plots to compare quantiles of each test set to the similarity within the training set (Figure 3). The pattern of results varied across the different similarity measures. Levenshtein similarities, a measure of word similarity regardless of semantic content, for the neurologist and non-neurologist notes diverged from the training set distribution in the upper quantiles, indicating lower maximum similarity values. This was true for both the kernels and contexts. Cosine similarities, a measure of semantic similarity, showed that kernels had slightly higher similarity values around the median for all 3 test sets compared to the training set, but similar values in the tails of all distributions. In contrast, cosine similarity of contexts showed clear shifts of the entire distributions, with the magnitude of shift increasing stepwise from epileptologists to neurologists to non-neurologists. This indicates that the most consistent textual difference across note categories was in the semantic content of the context surrounding the target kernels.

### Evaluating out-of-institution generalization performance

**Figure 4.**
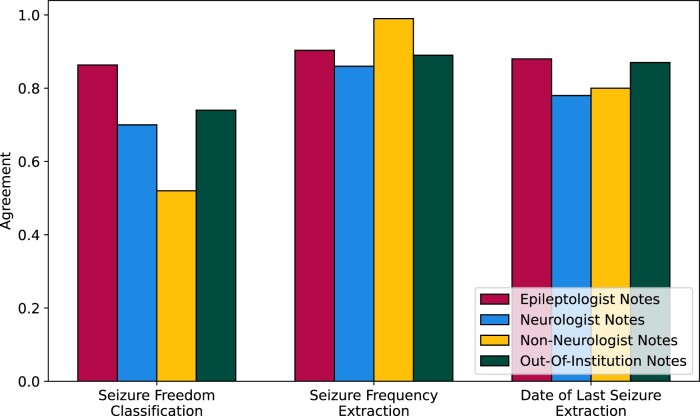
Generalization of our NLP pipeline on epileptologist, neurologist, and non-neurologist notes from the University of Pennsylvania, and on epileptologist notes from the University of Michigan, measured as agreement between algorithm predictions and human-annotated ground truth.

Epilepsy notes from the University of Michigan had similar distribution of annotations to the Penn epileptologist notes: 23% seizure free, 63% had recent seizures, and 14% were unclassifiable. Thirty-three percent of notes had seizure frequency text and 51% had date of last seizure text. From manual review, 85% of the passages isolated by our preprocessing contained all of the correct information. Overall, our pipeline’s classification performance decreased by 0.12 agreement when making predictions on Michigan epilepsy notes (Figure 4). Our pipeline’s ability to extract seizure frequency and date of last seizure texts remained constant between institutions, achieving 0.89 and 0.87 agreement on Michigan notes, respectively, versus 0.90 and 0.88 agreement on Penn epileptologist notes.

## DISCUSSION

In this study, we evaluated the generalization performance of our NLP pipeline. We tested its ability to accurately extract seizure outcome measures in new datasets without additional finetuning. We found that it was less accurate in classifying seizure freedom in notes written by non-epilepsy specialists. However, it was able to extract seizure frequency text and date of last seizure text from epilepsy notes from the University of Michigan with agreement to the ground truth comparable to epileptologist notes from Penn. Declines in performance in notes written by nonspecialists in epilepsy were associated with different distributions of ground-truth answers across note categories, and with increasing semantic and syntactic distance from the training set.

Overall, these findings indicate that our models, which were trained and validated on clinical notes from a particular medical subspecialty, performed less well on notes written by providers outside that subspecialty, even when those notes addressed the same medical condition. The observation that our models generalized relatively well to subspecialist notes from a different institution suggests that the major driver of performance was the content of notes written by providers from different specialties. These changes in performance are not unseen in previous studies. Machine learning algorithms perform worse in new contexts due to distribution shifts when the underlying distribution of its training data becomes too different from the testing distribution,^24–27^ including in clinical domains.^28–32^ Pretrained Transformer models are more robust to these shifts, but still show generalization gaps and variable reactions to previously unseen data.^33^^,^^34^ Adding to these prior works, our study is among the first to evaluate the generalizability of such algorithms in the clinical domain using multiple sets of expert-annotated real-world data with tangible and impactful applications,^15^ rather than data prepared for competitions or benchmarking.

We analyzed textual similarities on 2 fronts using the Levenshtein and cosine similarities, and applied these measures separately to the target text in the note for the pipeline to focus on, and the surrounding content. Decreases in similarities between providers then influenced changes in model performance. Other groups have found similar but have primarily used only the cosine distance on documents as a whole to measure semantic similarity; furthermore, few groups have applied such methods to clinical problems, and none to epilepsy. For example, Khambete et al^35^ found that model performance in classifying clinical diagnosis sentiment had a significant linear negative correlation with median semantic cosine distance. More studies have exploited this notion of cosine similarity to improve model performance or in novel applications: Chang et al^36^ used Explicit Semantic Analysis^37^ with Wikipedia to generate semantic representations of labels and documents for dataless classification. Similarly, Haj-Yahia et al^38^ and Schopf et al^39^ semantically matched text to classification labels for unsupervised text classification. Meanwhile, Kongwudhikunakorn et al^40^ used word embeddings and the Word Mover’s Distance^41^ to accurately cluster documents.

Methods to improve generalizability are numerous, but have sparsely been applied in the clinical domain. One method to combat distribution shift is to expand the training distribution by incorporating multiple training datasets;^42^^,^^43^ Hendrycks et al^33^ reasoned that pretrained transformers are already more robust to distribution shifts than previous neural models because their pretraining already covered diverse data. Santus et al^28^ found that models trained on reports from 4 hospitals outperformed models trained on only one. Similarly, Khambete et al^35^ found that models trained with sentences from multiple medical specialties outperformed models trained from just one. Other studies reframe the problem, improving generalization performance even in the absence of training data. For example, Yin et al^44^ proposed text classification as a textual entailment problem, where the question is converted into a statement (ie, “Does the patient have recent seizures” becomes “The patient is having recent seizures”) and the model is tasked with identifying if that statement is implied by or is contradicted by the main paragraph. Similarly, Halder et al^45^ redesigned generic text classification as repeated true/false classification where a model receives each label separately and in sequence, and must determine if that label is true given the paragraph. Alcoforado et al^46^ used topic modeling for text classification by finding what topics were in a given paragraph, and modeling how those topics entail specific labels.

Our study has some limitations. Our approach is agnostic to the type of seizure and provides only one of each outcome measure per note, potentially missing or confounding information from patients with multiple semiologies. From a clinical perspective, seizures also have varying severities, necessitating different response. For example, bilateral tonic-clonic convulsions may require trips to the hospital, whereas a simple focal aware seizure may not. Our notes and models were affected by copy-forwarded information, where a note author will copy previous notes into the current note, potentially introducing outdated or directly contradictory information. Our evaluation on notes from the University of Michigan also used a single seed to minimize the need for manual review and therefore lacks the statistical rigor of our other analyses. Furthermore, we required some additional tuning of the preprocessing steps when adapting our pipeline to the notes from the University of Michigan, which may limit the ease or speed at which it can be deployed at other institutions with a significantly different note format from ours. Our investigation was focused on the immediate clinical utility of our pipeline in new care contexts and at new institutions, and as such we did not perform a more in-depth and technical analysis by comparing our methods with those of other few-shot and zero-shot models and approaches.

## CONCLUSIONS

In conclusion, on nonepileptologist within-institution notes, our NLP pipeline experienced decreased performance when classifying seizure freedom, and variable performance when extracting seizure frequency text and date of last seizure text on certain subsets. Our text extraction algorithm required some adaptation to identify the relevant text in out-of-institution epileptologist notes. However, after identifying the correct text, the decrease in classification performance was more modest; performance on text extraction performance did not change on this dataset. Generalization performance was affected by differences between each dataset and the original training and validation datasets. We plan to improve our overall generalization performance in the future to allow for large-scale multicenter studies.

## Data Availability

Our code is available at https://github.com/penn-cnt/generalization_of_nlp_in_clinical_contexts. Our models are available in Huggingface Hub at https://huggingface.co/CNT-UPenn.
